# The Association between Pediatric NAFLD and Common Genetic Variants

**DOI:** 10.3390/children4060049

**Published:** 2017-06-18

**Authors:** Giuseppina Rosaria Umano, Mariangela Martino, Nicola Santoro

**Affiliations:** 1 Department of Pediatrics, Yale University, New Haven, CT 06520, USA; giuseppina.umano@yale.edu (G.R.U.); mari_martino@virgilio.it (M.M.); 2 Dipartimento della Donna, del Bambino, di Vhirurgia Generale e Specialistica, Universita’ della Campania Luigi Vanvitelli, 80138 Napoli, Italy; 3 Dipartimento di Medicina V. Tiberio, Universita’ del Molise, 86100 Campobasso, Italy

**Keywords:** NAFLD, children, genetics

## Abstract

Non-alcoholic fatty liver disease (NAFLD) is one of the most common complications of obesity. Several studies have shown that genetic predisposition probably plays an important role in its pathogenesis. In fact, in the last few years a large number of genetic studies have provided compelling evidence that some gene variants, especially those in genes encoding proteins regulating lipid metabolism, are associated with intra-hepatic fat accumulation. Here we provide a comprehensive review of the gene variants that have affected the natural history of the disease.

## 1. Introduction

Non-alcoholic fatty liver disease (NAFLD) is a complex disease arising from both genetic and environmental factors. It encompasses a wide spectrum of liver disease ranging from simple steatosis and steatohepatitis (NASH) to fibrosis and cirrhosis. Obesity and insulin resistance play a pivotal role in the natural history of the disease [1,2,3,4]. Nevertheless, the observation that not all obese patients develop a fatty liver suggests that a genetic background underlies this condition. Moreover, the importance of genetics is highlighted by twin studies [5,6] and ethnic differences in disease rates. In fact, its prevalence in Hispanics is higher than in individuals of European and African American descents [7,8]. Based on these findings, a large number of genome-wide association studies (GWAS) and single candidate gene studies have been conducted. The aim of this review is to provide an overview of the most common genetic variants associated with pediatric NAFLD.

## 2. An Overview of the Genetic Variants Associated with NAFLD Identified by GWAS 

The first GWAS for NAFLD was conducted by Romeo and coworkers, which reported a strong association between the Patatin-like phospholipase domain-containing 3 (*PNPLA3*) rs738409 gene variant and intra-hepatic fat content in the Dallas Heart Study [9]. Speliotes et al. reported that, along with *PNPLA3,* variants in Protein Phosphatase 1 Regulatory Subunit 3B (*PPP1R3B*), Glucokinase Regulatory Protein (*GCKR*), Neurocan (*NCAN*) and Lysophospholipase Like 1 (*LYPLAL1*) genes were also associated with NAFLD [10]. 

Later, Adams et al. showed an association between biopsy-proven NAFLD and two Single Nucleotide Polymorphisms (SNPs) in Gc Protein (*GC*) gene and lymphocyte cytosolic protein 1 (*LCP1*) gene in adolescents [11]. The *GC* gene encodes for vitamin D-binding protein, the main serum carrier for Vitamin D. The rs222054 SNP reduces the hepatic expression of the protein and affects the Vitamin D serum levels. *LCP1* is an actin-binding protein mainly expressed in hematopoietic cells. It is involved in leukocyte activation and tumor cell proliferation. It also seems to be expressed in the liver, where the presence of the rs7324845 variant leads to over-expression of the protein. Despite this evidence, the role of these two SNPs in NAFLD pathogenesis is not clear. Interestingly, the authors reported two other significant variants in two genes expressed in brain tissue—Phospholipid Phosphatase Related 4 (*LPPR4*) rs12743824 and Solute Carrier Family 38 Member 8 (*SLC38A8*) rs11864146—[11], but replication and functional data to validate these findings to date have not been provided. 

More recently, a GWAS conducted on patients affected by Alcoholic Liver Disease (ALD) reported an association between Membrane Bound O-Acyltransferase Domain Containing 7 (*MBOAT7*) polymorphisms and liver fibrosis. The authors identified two SNPs (rs626283 and rs641738) in high linkage disequilibrium that were associated with severe liver damage. The rs626283 polymorphism showed the strongest association [12]. The gene encodes for a lysophosphatidylinositol acyltransferase, which catalyzes acyl-chain remodeling of phosphatidylinositols. The protein is highly expressed in the liver, specifically in endoplasmic reticulum and in mitochondria-associated membranes. The variant consists of a C-to-T substitution and leads to a down-regulation of *MBOAT7* expression. Mancina et al. reported an association between the rs641738 variant in the *MBOAT7* gene and both the occurrence and progression of NAFLD. The minor allele (T) was significantly associated with high fat content only in African Americans, but not in European Americans and Hispanics. Moreover, the variant showed an additive effect with *PNPLA3* and Transmembrane 6 Superfamily Member 2 (*TM6SF2*) SNPs in determining the risk for steatosis. These findings suggest that an impaired protein activity enhances the arachidonic acid concentration leading to fat deposition and inflammation [13].

So far, only a few variants have been reproducibly associated with NAFLD in pediatric populations: *PNPLA3* rs738409*, GCKR* rs1260326, and *TM6SF2* rs58542926 (Table 1).

### 2.1. PNPLA 3

The *PNPLA3* gene encodes for a 481 amino acid transmembrane protein called adiponutrin [14]. The protein is expressed in the liver and adipose tissue [15] and its expression is influenced by food assumption, in particular by carbohydrate and lipids intake [16]. It exerts several activities: lipids hydrolysis [15], acyl-CoA thioesterase [17], and lysophosphatidic acid acyltransferase activity [18].

In a GWAS, Romeo and colleagues first described the non-synonymous variant I148M of *PNPLA3* as the strongest determinant of liver fat deposition, independent of ethnicity and other known risk factors for steatosis [9]. The variant encodes an isoleucine to methionine substitution in a high conserved residue [9]. Later, this association was confirmed in children [19,20]. The mechanism by which the substitution induces liver fat accumulation is still debated. Recently, Li and colleagues reported that the *PNPLA3* I148M variant presents an increased lipogenic activity, an impaired triglycerides hydrolysis and a depletion of long-chain polyunsaturated fatty acid (PUFA) in transgenic mice [21]. Other studies suggest that the loss of triglycerides hydrolysis might be the major cause of fat liver accumulation. In fact, the substitution modifies the tridimensional structure of the catalytic domain, reducing the substrate access and then triglycerides hydrolysis [17]. 

Several evidences show that environmental factors interact with this variant, leading to more severe liver damage. It has been reported that both obese and overweight children carrying the 148M allele present significantly higher serum liver enzymes levels compared to non-carriers [22,23,24]. A high intake of omega 6 PUFA and a high intake of carbohydrates seem to interfere with adiponutrin action and favor intra-hepatic fat accumulation when the minor allele of the rs738409 variant is present [25]. On the other hand, subjects carrying the minor allele for this variant seem to be more susceptible to the beneficial effects of weight loss on intra-hepatic fat [26,27].

Interestingly, more recently, another *PNPLA3* polymorphism has been described. Donati and coworkers reported an association between NAFLD and the E434K variant. Functional analysis revealed that this variant influences the *PNPLA3* expression by increasing adiponutrin levels [28].

### 2.2. GCKR

GWAS have linked the *GCKR* rs1260326 polymorphism to hypertriglyceridemia, hyperglycemia and NAFLD [29,30]. The gene encodes for the glucokinase regulatory protein that bids the glucokinase protein (GK) in the nucleus, making GK inactive. GK is involved in the first step of glycolysis, catalyzing the phosphorylation of glucose to glucose-6-phosphate (G6P) [31]. The rs1260326 variant encodes for a proline to leucine substitution at the 446 position (P446L), resulting in a loss of the affinity of the *GCKR* for the GK. As a consequence, more GK is available in cytoplasm to convert glucose in glucose 6 phosphate (G6P). The increased production of G6P results in an increased rate of glycolysis, which in turn leads to an increased production of malonyl-CoA, the precursor of de novo lipogenesis [32]. This variant has been associated with NAFLD not only in adults [10], but also in children [33]. In particular, an association between 446L variant and steatosis, high triglycerides, and increased large Very Low Density Lipoprotein (VLDL) has been reported in a population of obese children [33,34,35]. Moreover, it has been shown through stable isotopes studies that the mechanism by which this variant determines intra-hepatic fat accumulation in obese adolescents is indeed an increased hepatic de novo lipogenesis [34]. Later, similar findings came from Lin et al. that evaluated several genetic variants in an Asian pediatric population [35]. 

### 2.3. TM6SF2

In 2014, an exome-wide association study reported that the *TM6SF2* rs58542926 variant was associated with high hepatic triglycerides content and elevated liver enzymes serum levels in adults enrolled in the Dallas Heart Study [36]. The *TM6SF2* gene is highly expressed in the liver, kidney and small intestine. It encodes for a transmembrane protein that regulates the normal VLDL secretion by the liver. The E167K variant is characterized by a change of a glutamate to a lysine at the codon 167. The substitution increases the degradation of the protein, reducing the VLDL secretion by about 50% in the subject carrying the 167K allele [36]. In a large obese pediatric population, children with at least one minor allele (T) more frequently showed NAFLD, higher Alanine Transaminase (ALT) and lower non-High Density Lipoprotein (non-HDL) cholesterol serum levels, even after adjustment for anthropometrical parameters and the *PNPLA3* I148M genotype [37]. The elevation of liver enzymes suggested a role in worsening the liver damage [37]. This finding was then confirmed in several clinical trials [38,39,40]. Goffredo et al. have shown that along with NAFLD, obese youth carrying the minor allele for the *TM6SF2* rs58542926 variant also have lower plasma concentrations of very large VLDL, therefore confirming the animal data in humans, suggesting an impairment of the VLDL secretion in these patients. Moreover, it has been reported that the *PNPLA3* rs738409, *GCKR* rs1260326 and *TM6SF2* rs58542926 have an additive effect of hepatic fat deposition in a multiethnic cohort of obese youth [40]. Interestingly, statin use resulted effective in reducing NASH prevalence in adults carrying the minor allele for the *TM6SF2* rs58542926 variant [41]. 

## 3. Genetic Variants Identified in Candidate Gene Studies

Unlike the GWAS, the candidate genes approach is hypothesis driven and evaluates the association between a single gene and a phenotype. It differs from GWAS approach as the latter is not hypothesis driven and examines several common genome variations to assess associations. The major limitations of the candidate gene approach is mainly represented by the limited reproducibility of the data. Despite that, so far several genetic variants have been associated with NAFLD through these studies. In particular, variants in genes involved in regulation of glucose and lipid metabolism have been evaluated.

In 2010, Dongiovanni et al. reported that two variants affecting the action of insulin receptor—Lys121Gln in Ectonucleotide Pyrophosphatase/Phosphodiesterase 1 (*ENPP1/PC-1*) and the Gly972Arg in Insulin Receptor Substrate 1 (*IRS-1*) genes—were significantly associated with NAFLD and the presence of fibrosis in a population of obese adults [42].

Contrasting results came from studies on the adiponectin gene (*ADIPOQ*) polymorphisms. Adiponectin enhances hepatic insulin sensitivity, promotes the free fatty acids oxidation, and reduces the proliferation of smooth muscle cell in vessels [43,44]. Three polymorphisms in the *ADIPOQ* gene have been described (45G > T, 276G > T, and 11377C > G) as associated with lower adiponectin plasma levels. It has been shown that these polymorphisms positively correlate with the presence and severity of steatosis, NASH and fibrosis [45]. Later, these findings were confirmed in Japanese [46] and Taiwanese adult populations [47], while it was not confirmed in a Chinese population [48]. Moreover, a recent meta-analyses found no association between the 45TG *ADIPOQ* variant and NAFLD [49]. 

Some investigators sought to find an association between some variants in the gene encoding for Leptin (*LEP*) and the Leptin receptor (*LEPR*). In fact, the *LEPR* rs3790433 variant has been previously associated with insulin resistance and metabolic syndrome features [50]. Therefore, *LEPR* gene polymorphisms have been analyzed in subjects with NAFLD. The variant G3057A in the *LEPR* gene was more prevalent in Chinese diabetic adults with NAFLD [51]. Moreover, Zain et al. found an association between two different SNPs (rs1137100 and rs1137101) in the *LEPR* gene and NAFLD occurrence among different Asian ethnic groups [52]. 

Contrasting results have been also shown regarding the influence of Peroxisome proliferator-activated receptor-alpha (*PPARα*) and Peroxisome proliferator-activated receptor-gamma (*PPARγ*) polymorphisms in NAFLD pathogenesis. *PPARα* encodes for a nuclear receptors superfamily, and it is highly expressed in tissues metabolizing fatty acids [53]. *PPARγ* encodes for a transcription factor, and drives adipose tissue differentiation, Free Fatty Acids (FFAs) uptake and storage [54]. Because the two factors are involved in lipid metabolism, it was suggested that their loss-of-function could play a role in fatty liver deposition. Chen et al. reported that the *PPARα* Val227Ala variant was associated with a significant NAFLD risk, being the Val227 isoform more prevalent in NAFLD patients [55]. Furthermore, the Lys162Val variant of the *PPARα* gene was associated with fatty liver and liver damage in Brazilian adults, while the Pro12Ala variant of the *PPARγ* gene showed a protective effect against NAFLD/NASH [56]. Later, the group of Dongiovanni reported a lack of association between both the Lys162Val variant in the *PPARα* gene and the Pro12Ala variant in the *PPARγ* gene with biopsy-proven NAFLD and NASH [57].

The *PPARGC1A* gene encodes for peroxisome proliferator-activated receptor γ coactivator 1α, which is involved in mitochondrial biogenesis, oxidative phosphorylation, and insulin resistance [58,59]. Yoneda et al. investigated the association between 15 SNPs of the *PPARGC1A* gene and NAFLD. The authors reported that the rs2290602 SNP was strongly associated with NAFLD and NASH [60]. Moreover, the variant was shown to be associated with liver enzymes serum levels in Taiwanese obese children [61]. However, this association could not be confirmed in a Chinese population [62]. 

Another gene associated with NAFLD pathogenesis is the Apolipoprotein C3 (*APOC3*) gene. Two SNPs (rs2854116 and rs2854117) in high linkage disequilibrium within the *APOC3* gene have been associated with hypertriglyceridemia and NAFLD [63,64] The first variant encodes for a substitution of threonine to cysteine at 455 position, and the second drives a cysteine to threonine change at 482 residue. Both of them lead to higher plasma *APOC3* concentration, enhancing the lipoprotein lipase inhibition, and resulting in high chylomicron remnants and plasma levels [64]. On the basis of these studies, Petersen et al. evaluated the role of *APOC3* polymorphisms in NAFLD in a population of Asian-Indian men [65]. The authors described an association between these SNPs and both liver fat content and insulin resistance in lean Asian-Indian men. Moreover, *APOC3* variants showed a joint effect with the *PNPLA3* variant leading to higher fat deposition in the liver. To date, the literature displays contrasting results about the association between this SNP and NAFLD. In a Chinese population study, the authors confirmed that association [66], but several more recent studies could not replicate those findings [67,68]. The reason for these discrepancies may be that Peterson et al. enrolled subjects that had no features of metabolic syndrome and obesity, while the studies that found no association included obese or overweight subjects. 

Another putative actor in the complex scenario of genetic predisposition of NAFLD might be the Microsomal Triglyceride Transfer Protein (*MTTP*) gene. *MTTP* encodes the microsomal transfer protein (MTP), which is expressed in the liver and small intestine. The protein transfers neutral lipids (as triglycerides and cholesterol esters) to the Apo B lipoprotein (chylomicrons, VLDL, and LDL) [69]. The protein’s loss of function has been linked to reduced lipoprotein plasma levels and NAFLD risk. The literature reports several SNPs affecting its activity, with contrasting results. A recent meta-analysis confirmed that the promoter −493G > T polymorphism might be associated with NAFLD risk [70,71,72,73,74]. The minor allele (GG) has been linked to an increased postprandial lipid production and larger VLDL circulating levels. The great amount of circulating lipids might increase the oxidative stress leading to Kupffer and stellate cells activation, and then to inflammation [72]. In 2015, a cross-sectional study investigated five different *MTTP* SNPs (−493G/T, E98D, I128T, N166S, and Q297H). The 297H variant was significantly associated with lower Low Density Lipoprotein (LDL-C) and non-HDL plasma levels and a greater NAFLD risk. The polymorphism seems to affect the tridimensional structure of the protein, reducing the ability of binding Apo-B [74]. 

Phosphatidylethanolamine *N*-methyltransferase catalyzes de novo synthesis of phosphatidylcholine in liver [75], and is encoded by the Phosphatidylethanolamine *N*-Methyltransferase (*PEMT*) gene. The *PEMT* non-synonymous variant V175M has been associated with fatty liver disease in humans and rats [75,76]. However, opposite results came from Romeo et al. that found a lack of association with fat liver content at Nuclear Magnetic Resonance-spectroscopy (NMR-spectroscopy) [77]. Moreover, Jun et al. found no difference in *PEMT* polymorphism frequencies in NAFLD and non-NAFLD patients [78]. 

Lipin1 (*LIPIN1*) gene encodes a phosphatase expressed both in liver and adipose tissue. The protein is involved in the metabolism of triacylglycerol and in adipogenesis. The rs13412852 TT genotype has been associated with lower rates of NAFLD, NASH, and fibrosis in children [79].

Finally, another gene that has been associated with fat liver content is liver-specific Fatty Acid Transport Protein 5 (*FATP5*). The rs56225452 polymorphism in *FATP5* gene has been positively related to NAFLD, hypertriglyceridemia and higher insulin resistance. In fact, patient homozygous for the A allele (minor allele) showed higher ALT serum levels and a higher prevalence of NAFLD. The protein is crucial for the uptake of fatty acid by the liver, and the variant compromises the promoter region of the gene, leading to lower expression [80]. 

## 4. Genes Involved in NAFLD Progression 

Both GWAS and candidate gene studies found several genes involved in the progression of liver damage. It is well known that NASH patients have an elevated expression of genes involved in stellate cells activation and fibrogenesis [81] (Table 2).

Oxidative stress due to reactive oxygen species (ROS) resulting from mitochondrial dysfunction, peroxisomal and microsomal fatty acid oxidation, and lipid peroxidation is a hallmark of NASH. Liver injury hampers the mitochondrial role in fatty acid oxidation, promotes steatosis and activates the cascade of ROS generation and subsequent endoplasmic reticulum (ER) stress [82].

In 2010, Chalasani et al. identified several variants conferring susceptibility to liver damage in women with NASH. Among them, the Farnesyl-Diphosphate Farnesyltransferase 1 (*FDFT1*) rs2645424 was significantly associated with the NAFLD Activity Score (NAS) [83]. The *FDFT1* gene encodes for squalene synthase, which catalyzes the production of squalene and modulates the cholesterol biosynthesis. It has been hypothesized that the protein gain of function might increase cholesterol biosynthesis in the liver. The association with liver damage was also replicated in a multiethnic cohort of obese youth with NAFLD [84].

*MBOAT7* is also known as lysophosphatidylinositol acyltransferase 1 (*LPIAT1*), which catalyzes acyl-chain remodeling of phosphatidylinositols (PIs). The *MBOAT7* rs626283 and rs641738 were first associated with severe liver damage in ALD [12]. Moreover, Krawczyk and coworkers reported an association between *MBOAT7* and fibrosis severity [85]. Recently, Vitasalo et al. reported that children carrying the minor allele showed higher ALT plasma levels compared to non-carriers. [86]. 

Also, *PNPLA3* is an important candidate in susceptibility to NASH. Recent studies have shown that this variant might play a role in the progression of histological damage both in adults [87,88,89,90] and children [91,92,93]. Valenti et al. observed that children homozygous for the G allele were at higher risk of hepatocytes ballooning, lobular inflammation and fibrosis, compared to the other genotype groups [91]. In addition, homozygosity for the G allele was associated with an increased pro-apoptotic gene expression, NASH and fibrosis in children independently of steatosis severity [92]. To date, the mechanism by which the variant could increase the hepatic fibrosis is still under debate. It has been hypothesized that *PNPLA3* could itself activate the hepatic stellate cells (HSC) via Hedgehog signaling pathway [94]. Alternatively, the loss of function in lipolysis could amplify lipoperoxidation, oxidative stress, and activation of both Kupffer cells and HSC [95]. 

Recently, it also has been suggested that the variant rs1260326 of *GCKR* might favor the progression of NAFLD towards NASH. In fact, Petta et al. reported an independent association between *GCKR* 446L and biopsy-proven NASH severity and fibrosis degree [96]. 

The *TM6SF2* gene rs58542926 variant is known to favor fat liver accumulation. Several studies also report an association with liver damage, both inflammation and fibrosis, in adults [38,39] and children [37,40].

Aberrations of insulin-signaling cascades and glucose homeostasis are crucial in NASH progression; therefore, it has been hypothesized that s121Gln in the *ENPP1/PC-1* gene and the 972Arg in the *IRS-1* reduce cell survival and affect lipogenesis, contributing to hepatocellular damage and fibrogenesis [42]. Moreover, SNPs in the adiponectin gene, 45TT and 276GT have been associated with severe liver disease, blunted postprandial adiponectin response, and an atherogenic postprandial lipoprotein profile [45].

Oxidative stress and lipid peroxidation are involved in enhancing tissue inflammation. Thus, several mediators have been investigated in NAFLD patients. In a GWAS, Kitamoto reported that genetic variants in the Sorting And Assembly Machinery Component 50 (*SAMM50*) and in the Parvin Beta (*PARVB*) predispose to NAFLD progression [97]. *SAMM50* encodes for a mitochondrial membrane protein (Sam50), thus the abnormal gene product might lead to a membrane instability and increased oxidative stress. The *PARVB* gene product belongs to the integrin proteins family; it is involved in cell-to-matrix interaction, so the authors suggested that the gene variant could be involved in fibrosis progression [97]. The *MTHFR* gene encodes for Methylenetetrahydrofolate reductase, a protein that catalyzes the methylation of homocysteine to methionine [98]. Hyperhomocysteinemia has been identified as a risk factor for liver oxidative stress and inflammation [99,100]. Polymorphisms leading to a loss of function of *MTHFR* gene product have been investigated in NAFLD patients. Saczy et al. reported a significant association between C677T and A1298C variants and NASH in a small Caucasian cohort [101], while other studies failed to replicate these findings [102,103]. Furthermore, it has been hypothesized that the 493G/T, E98D, I128T, N166S, and Q297H variants in the *MTTP* gene cause an increased postprandial lipid production, which in turn impairs the defense against the oxidative stress, leading to Kupffer and stellate cells activation, and then to inflammation [74].

Genetic variants in genes coding for pro-inflammatory mediators might predispose to disease progression. Cytokines are soluble molecules that are involved in intercellular communication and are produced by a wide variety of cells in the body, including most types of hepatic cells. They are involved in several diseases, as well as in the progression of liver damage. Increased levels of pro-inflammatory factors—i.e., tumor necrosis factor alpha (TNF-α), interleukin 1 beta (IL1-β), and interleukin 6 (IL-6)—may worsen the severity of liver damage. TNF-α increases insulin resistance through inhibiting the kinase activity of the insulin receptor [104]. High serum TNF-α levels have been linked to NAFLD [105,106] and NASH severity [80]. Valenti et al. described that the promoter variant -238 in the *TNF-α* gene is associated with NAFLD and lower TNF-α serum levels in an Italian cohort [105]. In 2007, Tokushige and coworkers reported that the -1301C and -863A promoter polymorphisms were more prevalent in NASH-affected patients, but no association was found for the other three variants (−857, −308, and −238) [107]. However, Wong et al. could not replicate this association [108]. In 2016, a meta-analysis concluded an association between promoter polymorphism at −238 position and NAFLD, but no clear association for the -308 promoter variant was found [109]. Also IL1-β and IL-6 exert a pro-inflammatory action, and their serum levels are higher in NASH patients [110]. Several studies report a significant association between Interleukin 1 beta (*IL1-β*) and Interleukin 6 (*IL-6*) polymorphisms and NASH [111,112]. Recently, four SPNs (*IL6* rs1049956, *IL6* rs1800795, *IL1β* rs16944, and *IL1β* rs1143634) have been analyzed in patients with biopsy-proven NASH [113]. Caucasian carriers of the *IL6* rs1049956C allele were at higher risk of NASH compared to non-carriers. Conversely, the *IL6* rs1800795 C allele was significantly associated with severe steatosis, but not with inflammation and fibrosis. The strongest polymorphism for NASH and fibrosis prediction was the *IL1β* rs1143634 TT genotype [113]. 

## 5. Conclusions

NAFLD is a major public health problem among obese children. The efforts in understanding its genetic background might help clinicians in preventing and treating this disease. However, although some genetic studies have clearly helped understanding its pathogenesis, these findings are far from being translated in clinical practice. Therefore, more effort is needed to better evaluate the genetic predisposition to NAFLD, the mechanisms through which gene variants lead to NAFLD, and how we could leverage this knowledge to provide a cure to kids with NAFLD.

## Figures and Tables

**Table 1:** SNPs reproducibly associated with pediatric fatty liver.

Gene	SNP	Function	Hepatic Fat	Circulating Lipids
*PNPLA3*	rs738409	Remodeling of lipid droplets	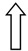	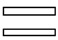
>*GCKR*	>rs1260360	>Modulation of hepatic lipogenesis	> 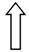	> 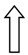
*TM6SF2*	rs58542926	Modulation lipoprotein secretion	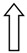	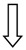

*GCKR*: Glucokinase Regulatory Protein; *PNPLA3*: Patatin-like phospholipase domain-containing 3; *TM6SF2*: Transmembrane 6 Superfamily Member 2; SNP: Single Nucleotide Polymorphism.

**Table 2:** Genes involved in NAFLD progression.

Gene	Association
*FDFT1*	rs2645424 could affect the progression toward fibrosis.
*MBOAT7*	rs626283 and rs641738 are two SNPs associated with fibrosis severity.
*PNPLA3*	The rs738409 variant is associated with an increased pro-apoptotic gene expression, NASH and fibrosis.
*GCKR*	rs1260326 SNP has an independent association with NAFLD, biopsy proven NASH and fibrosis.
*TM6SF2*	rs58542926 SNP is associated with NAFLD, NASH, and fibrosis.
*NNPP1/PC-1* *IRS-1*	The Lys121Gln variant in the *NNPP1/PC-1* gene and the Gly972Arg in the *IRS-1* gene are associated with fibrosis.
*ADIPOQ*	45G > T, 276G > T and 11377C > G are associated with lower adiponectin plasma levels, severity of steatosis, NASH, and fibrosis.
*SAMM50*	Sam50 polymorphisms might expose hepatic cells to higher oxidative stress.
*MTHFR*	C677T and A1298C variants are associated with NASH.
*MTTP*	−493G/T, E98D, I128T, N166S, and Q297H variants have been linked to an increased post prandial lipogenesisthat enhances the oxidative stress leading to Kupffer and stellate cells activation and then to inflammation.
*TNF-α*	−1301C and -863A promoter polymorphisms are more prevalent in NASH.
*IL 1-β* and *IL-6*	*IL 1-β* rs16944, *IL 1-β* rs1143634 and *IL-6* rs1049956 has been investigated in patients with biopsy proven NASH.

NAFLD: Non-Alcoholic Fatty Liver Disease; NASH: Non-Alcoholic Steatohepatitis.
